# Visual prognosis, intraocular pressure control and complications in phacomorphic glaucoma following manual small incision cataract surgery

**DOI:** 10.4103/0301-4738.64136

**Published:** 2010

**Authors:** R Ramakrishanan, Devendra Maheshwari, Mohideen Abdul Kader, Rita Singh, Neelam Pawar, M Jayahar Bharathi

**Affiliations:** Aravind-Ziess Centre for Excellence in Glaucoma, Aravind Eye Hospital & Postgraduate Institute of Ophthalmology, Tirunelveli, Tamil Nadu - 627 001, India

**Keywords:** Intraocular pressure, manual small incision cataract surgery, phacomorphic glaucoma

## Abstract

**Aims:**

To evaluate intraocular pressure (IOP) control, visual prognosis and complications following manual small incision cataract surgery among eyes with phacomorphic glaucoma.

**Materials and Methods:**

This prospective, non-randomized interventional consecutive case series included all patients with phacomorphic glaucoma who presented to a tertiary eye care referral center in South India between March 2006 and April 2007. All patients underwent slit-lamp bio-microscopy, applanation tonometry and gonioscopy of the other eye to rule out angle closure. Small incision cataract surgery with intraocular lens implantation was performed in all affected eyes. Complete ophthalmic examination was done at each follow-up visit.

**Results:**

A total of 74 eyes with phacomorphic glaucoma were included in this study. The preoperative mean IOP was 38.4±14.3 mmHg and mean IOP at last follow-up was 12.7±2.4 mmHg. There was a statistically significant difference between IOP at presentation and IOP at last follow-up (*P*< 0.001). None of the eyes required long-term antiglaucoma medication. No significant intraoperative complications were noted. The final postoperative best corrected visual acuity was 20/40 or better in 51 patients. Eighteen eyes had corneal edema and 36 eyes had anterior chamber inflammation. Both conditions resolved with standard medical therapy.

**Conclusion:**

Manual small incision cataract surgery is safe and effective in controlling IOP and achieving good functional visual acuity with minimal complications in the management of phacomorphic glaucoma in developing countries.

Cataract has been documented to be the most significant cause of bilateral blindness both in India as well as on a global scale.[1‐4] It has been estimated that there are about 12.5 million blind people in India, with 50 to 80% of this group due to cataract.[2‐5] Phacomorphic glaucoma is a common entity in developing countries like India due to lack of awareness of cataract and delayed surgical interventional removal. This is mainly due to the general misbelief that cataract should be mature at the time of surgery to avoid complications.[6] Lack of need for better vision, concurrent systemic disease, old age and economic constraints are among other reasons for patients not receiving treatment.[6] In India the incidence of intumescent cataract leading to phacomorphic glaucoma is more when compare to European population.[7]

Cataract extraction remains the only definitive treatment for an intumescent cataract.[8] Cataract surgery in phacomorphic glaucoma poses several challenges: the high intraocular pressure (IOP) increases the risk of expulsive hemorrhage, positive pressure and there is often zonular dialysis which makes surgery technically more difficult. Manual small incision cataract surgery (MSICS) is popular in developing countries as it is inexpensive compared to instrumental phacoemulsification and allows high-volume cataract surgery without compromising quality of medical care.[9‐12] Our study aimed to evaluate the visual prognosis, IOP control and complications following MSICS in the management of patients with phacomorphic glaucoma.

## Materials and Methods

This non-randomized, interventional case series study was conducted at the Aravind Eye Hospital and Postgraduate Institute of Ophthalmology, Tirunelveli, India and was approved by the hospital's institutional review board. All patients diagnosed with phacomorphic glaucoma between March 2006 and April 2007 were included in the study. Phacomorphic glaucomas were diagnosed by subjective complaints of acute pain and redness associated with objective signs such as presence of corneal edema, shallow anterior chamber, an intumescent cataractous lens and IOP above 21 mmHg.[13] The preoperative assessment included slit-lamp examination, applanation tonometry, gonioscopy of other eye to rule out angle closure and B-scan ultra-sonography to exclude posterior segment pathology. Gonioscopy of other eye was done to rule out angle closure glaucoma and all those in whom the fellow eye had narrow angle and raised IOP were excluded from the study. All patients were treated with topical beta blockers, antibiotic steroid drops, oral acetazolamide and oral glycerol. If the IOP was more than 45 mmHg, intravenous mannitol was given. All surgeries were performed by a single surgeon.

Preoperatively intravenous mannitol 20% (1-2 g/kg body weight) was given in all patients. Surgeries were performed under retrobulbar anesthesia with facial block. After superior fornix-based conjunctival flap, a partial thickness 6.0–6.5 mm scleral incision was made 2 mm behind the limbus and a scleral tunnel was extended 1 mm into clear cornea. An additional paracentesis was made at the 10 o'clock position. The anterior chamber was filled with an air bubble and 0.1 ml of 0.06% trypan blue (Auroblue, Aurolab, India) was injected under the air bubble. After several seconds, viscoelastic (Aurovisc, Aurolab, India) was used to displace the air bubble. The anterior chamber was entered with a 3.2 mm keratome. Anterior chamber was deepened with viscoelastic and continuous curvilinear capsulorrhexis (CCC) was made in the anterior capsule using a bent 26G needle cystitome. The capsular bag was inflated with viscoelastic and the CCC was completed using an utrata capsulorrhexis forceps. A Sinskey hook was used to hook out one pole of the nucleus outside the capsular bag and the rest of the nucleus was rotated out anticlockwise or clockwise into the anterior chamber. The nucleus was extracted out of the eye using an irrigating vectis (Indo-German, India). The hard brown cataract was prolapsed by using a cyclodialysis spatula which supports and acts as a fulcrum to prolapse nucleus avoiding stress on zonules. After aspiration of the remaining cortex, a 6-mm optic polymethyl methacrylate posterior chamber intraocular lens (Aurolab, India) was implanted into the capsular bag. The viscoelastic material was aspirated and both the wound and paracentesis were hydrated with balanced salt solution. The conjunctival flap was opposed using a forceps fitted to bipolar diathermy. Subconjuctival injection of garamicin and dexamethasone was given.

Postoperatively, patients were treated with topical antibiotics and steroids for the next six to eight weeks. On follow-up at the 90^th^ postoperative day, patients underwent an independent ophthalmic examination by a trained ophthalmologist. All statistical analyses were performed using SPSS Version 16.0 (SPSS Inc, Chicago, Illinois, USA). Chi Square tests were used to compare the results of categorical variables, and Student t tests used to compare means, with *P* value 0.05 considered statistically significant.

## Results

The demographic details of the patients included in the study are presented in Table 1. A total of 74 patients of mean age 66.61±9.2 years with phacomorphic glaucoma were analyzed during the study period of one year. Out of 74 patients who underwent posterior chamber intraocular lens (PCIOL) implantation, one patient had zonular dialysis, which was stabilized by a capsular tension ring, five had posterior capsule rupture and 10 patients had intraoperative shallowing of anterior chamber due to positive pressure. On the first postoperative day, there was severe corneal edema in 18 (24%) patients, 36 (48%) had severe iritis with fibrin membrane, two had blood clot. Blood clot resolved with medical treatment over a period of one week. The fibrin membrane and corneal edema resolved with topical medications over the next few days. The preoperative IOP and the best corrected visual acuity (BCVA) at last follow-up are summarized in Table 2. Fifty-one (68%) patients attained 20/40 or better vision, of which 30 (58%) had preoperative IOP between 25-40 mm Hg and 21 (41%) had IOP between 41-55 mm Hg. There was no association between preoperative IOP and postoperative BCVA (*P*=0.361).

**Table 1:** Pre-operative demographic characteristics of the study population

Demographics	Number of patients (%)
Age (years)	
Mean (± SD)	66.6 (± 9.2)
Range	40 - 89
Gender	
Male	22 (29)
Female	52 (70)
Status of fellow eye	
Immature cataract	42 (56)
Mature cataract	5 (6)
Aphakia	3 (4)
Pseudophakia	17 (22)
Clear lens	7 (9)
Duration of symptoms (Days)	
0-10	61 (82)
11-20 days	12 (16)
21-30 days	1 (1)
Preoperative IOP(mmHg)	
25-40 mmHg	44 (58)
41-55 mmHg	27 (36)
56-70 mmHg	3 (4)
BCVA	
Accurate projection of rays	66 (89)
Inaccurate projection of rays	8 (10)
Systemic medical conditions	
Nil	55 (74)
Diabetes	5 (6)
Hypertension	10 (13)
Diabetes and hypertension	4 (5)

**Table 2:** Comparative analysis of patients with preoperative IOP and postoperative visual acuity at third month follow-up visit

Postoperative BCVA	Preoperative IOP		
	25 - 40 mmHg	41 - 55 mmHg	56 - 70 mmHg
20/20-20/40	30	21	-
20/60-20/200	12	3	-
<20/ 200	1	1	1
HM, PL	1	2	2

BCVA: Best corrected visual acuity, IOP: Intraocular pressure, HM: Hand movement, PL: Perception of light

The duration of symptoms and the BCVA at last follow-up are summarized in Table 3. Forty-three (70%) patients had 20/40 vision when duration of symptom was less than 10 days, seven (58%) had 20/40 or better vision when duration of symptom was 11 days to 20 days. Comparing the patients with symptoms for less than 10 days against patients with symptoms of 10 days or longer, there was significant association between duration of symptoms and postoperative BCVA (Chi square test *P*< 0.008).

**Table 3:** Effect of duration of symptoms on visual outcome

Postoperative BCVA	Duration of symptoms [Number (%)]		
	0-10 days	11-20 days	21-30 days
20/20-20/40	43	7	1
20/60-20/200	12	3	-
<20/200	2	0	-
HM, PL	4	2	-

BCVA: Best corrected visual acuity, HM: Hand movement, PL: Perception of light

The mean IOP on the final visit was 12.7±2.4 mmHg (range, 5–20) without the use of antiglaucoma medications. At the last follow-up visit by the end of the third month, the BCVA was 20/40 or better in 51 patients (68%), 20/60 to 20/200 in 15 patients (20%), <20/200 in two patients and hand movement (HM) in six patients.

## Discussion

A shallow anterior chamber with high IOP due to phacomorphic glaucoma is a common occurrence in developing countries. Extra-capsular cataract extraction (ECCE) requires a large incision in a globe with very high IOP, which increases the risk of sight-threatening complications.[9,11] MSICS with trypan blue staining of anterior capsule has an advantage over ECCE and phacoemulsification. It has been shown that MSICS gives better uncorrected vision compared to ECCE due to higher postoperative astigmatism in ECCE.[9] Ruit *et al*., reported that both phacoemulsification and MSICS achieved excellent visual outcomes with low complication rates. MSICS may be the more appropriate surgical procedure for the treatment of advanced cataracts in the developing world.[21,22] Venkatesh *et al*. have reported MSICS to be safe and effective for management of phacolytic glaucoma.[23] Phacoemulsification is difficult in phacomorphic glaucoma because there is an increased risk shallow chamber, iris prolapse, peripheral capsulorrhexis tears; the risk endothelial cell loss is greater because of the close proximity of the phaco tip during nucleus emulsification and the reduced endothelial reserve in these patients.[19] In such cases Chang *et al*., have advised pars plana vitreous tap to expand the anterior segment which helps to deepen anterior chamber and permits successful completion of capsulectomy and cataract removal.[24] Dada *et al*., have also reported the use of a sutureless, small-gauge, pars plana partial-core vitrectomy as an effective technique to overcome these problems but the limitations of this technique are that direct visual control is often not possible because of the dense cataracts and there is a small risk of retinal detachment, as reported after small-gauge vitrectomy for various posterior segment disorders.[19] In contrast, MSICS does not require expensive equipment like phacoemulsification and the anterior chamber is more stable due to the shelving scleral wound along with minimal surgical-related complications.

In our study, females (70%) predominate male (29%), it is possible due to socioeconomic constraints.

Three months postoperatively, 51 (68%) of our patients had good visual outcome with BCVA of 20/40 or better and, 20/60 to 20/200 in 15 (20%) patients. These visual outcome results are comparable with other studies of ECCE performed in phacomorphic glaucoma.[14,17] There was a significant association between duration of symptoms and postoperative BCVA. Fifty one patients had visual acuity better than 20/40, out of them significantly more number of patients had duration of onset of symptoms less than 10 days (84%; 43 of 51) compared to those with more than 10 days (16%; 8 of 51). Shorter the duration between onset of symptoms and surgery better the prognosis of visual acuity. The IOP on the final visit was less than 20 mmHg in all 74 patients without the use of anti-glaucoma medications, with a mean IOP of 12.7±2.4 mmHg. In all our cases, the IOP was controlled without the need for long-term anti-glaucoma medications. This result is similar to IOP control of other studies on ECCE performed for phacomorphic glaucoma.[14‐17] There was a statistically significant difference between IOP at presentation and IOP at last follow-up (Student t test *P* <0.0001). Two patients who had BCVA < 20/200 had preexisting diabetic retinopathy with macular edema. Out of six patients with HM, three had optic disc pallor, three had glaucomatous atrophy. This was likely secondary to the prolonged raised IOP associated with the phacomorphic glaucoma. The corneal edema and anterior chamber inflammation along with fibrinous reaction detected on the first postoperative day is not unusual considering the intense inflammation associated with phacomorphic glaucoma[19] and resolved with medical therapy.

MSICS is popular in developing countries as it is inexpensive, has a shorter learning curve compared to phacoemulsification and allows high-volume cataract surgery without compromising quality of medical care. In developing countries like India, phacomorphic glaucoma is not an uncommon presentation in the population. Our study has a few limitations being a non-randomized study design and of having a short follow-up period, however, we would wish to show that MSICS could be safe and effective in controlling IOP and attaining good functional visual recovery in the management of phacomorphic glaucoma with minimal complications in the developing world.
